# Weight loss and outcomes in subjects with progressive pulmonary fibrosis: data from the INBUILD trial

**DOI:** 10.1186/s12931-023-02371-z

**Published:** 2023-03-09

**Authors:** Michael Kreuter, Elisabeth Bendstrup, Stéphane Jouneau, Toby M. Maher, Yoshikazu Inoue, Corinna Miede, Dirk Lievens, Bruno Crestani

**Affiliations:** 1 Center for Interstitial and Rare Lung Diseases, Pneumology and Respiratory Care Medicine, Thoraxklinik, University of Heidelberg, and German Center for Lung Research, Heidelberg, Germany; 2 Center for Pulmonary Medicine, Departments of Pneumology, Mainz University Medical Center and of Pulmonary, Critical Care & Sleep Medicine, Marienhaus Clinic Mainz, Mainz, Germany; 3 Department of Respiratory Diseases and Allergy, Centre for Rare Lung Diseases, Aarhus University Hospital, Aarhus, Denmark; 4 Department of Respiratory Medicine, Competences Centre for Rare Pulmonary Diseases, CHU Rennes, University of Rennes, Rennes, France; 5 Keck School of Medicine, University of Southern California, Los Angeles, CA USA; 6 Clinical Research Center, National Hospital Organization Kinki-Chuo Chest Medical Center, Sakai City, Osaka Japan; 7 mainanalytics GmbH, Sulzbach (Taunus), Germany; 8 Boehringer Ingelheim International GmbH, Ingelheim am Rhein, Germany; 9 Pneumologie, Hôpital Bichat, Paris, France

**Keywords:** Interstitial lung disease, Pulmonary fibrosis, Forced vital capacity

## Abstract

**Background:**

Lower body mass index (BMI) and weight loss have been associated with worse outcomes in some studies in patients with pulmonary fibrosis. We analyzed outcomes in subgroups by BMI at baseline and associations between weight change and outcomes in subjects with progressive pulmonary fibrosis (PPF) in the INBUILD trial.

**Methods:**

Subjects with PPF other than idiopathic pulmonary fibrosis were randomized to receive nintedanib or placebo. In subgroups by BMI at baseline (< 25, ≥ 25 to < 30, ≥ 30 kg/m^2^), we analyzed the rate of decline in FVC (mL/year) over 52 weeks and time-to-event endpoints indicating disease progression over the whole trial. We used a joint modelling approach to assess associations between change in weight and the time-to-event endpoints.

**Results:**

Among 662 subjects, 28.4%, 36.6% and 35.0% had BMI < 25, ≥ 25 to < 30 and ≥ 30 kg/m^2^, respectively. The rate of decline in FVC over 52 weeks was numerically greater in subjects with baseline BMI < 25 than ≥ 25 to < 30 or ≥ 30 kg/m^2^ (nintedanib: − 123.4, − 83.3, − 46.9 mL/year, respectively; placebo: − 229.5; − 176.9; − 171.2 mL/year, respectively). No heterogeneity was detected in the effect of nintedanib on reducing the rate of FVC decline among these subgroups (interaction p = 0.83). In the placebo group, in subjects with baseline BMI < 25, ≥ 25 to < 30 and ≥ 30 kg/m^2^, respectively, 24.5%, 21.4% and 14.0% of subjects had an acute exacerbation or died, and 60.2%, 54.5% and 50.4% of subjects had ILD progression (absolute decline in FVC % predicted ≥ 10%) or died over the whole trial. The proportions of subjects with these events were similar or lower in subjects who received nintedanib versus placebo across the subgroups. Based on a joint modelling approach, over the whole trial, a 4 kg weight decrease corresponded to a 1.38-fold (95% CI 1.13, 1.68) increase in the risk of acute exacerbation or death. No association was detected between weight loss and the risk of ILD progression or the risk of ILD progression or death.

**Conclusions:**

In patients with PPF, lower BMI at baseline and weight loss may be associated with worse outcomes and measures to prevent weight loss may be required.

*Trial registration:* https://clinicaltrials.gov/ct2/show/NCT02999178.

**Supplementary Information:**

The online version contains supplementary material available at 10.1186/s12931-023-02371-z.

## Plain language summary

Patients with worsening fibrosis (scarring) of the lungs may lose weight. This study suggests that the course of disease may be worse in patients who lose weight. Measures to prevent weight loss may be needed in these patients.

**Supplementary Information:**

The online version contains supplementary material available at 10.1186/s12931-023-02371-z.

## Background

Patients with various forms of fibrosing interstitial lung disease (ILD) may develop progressive fibrosing ILD, more recently termed progressive pulmonary fibrosis (PPF), characterized by decline in lung function, increasing fibrosis on radiology, worsening symptoms and quality of life, and high mortality [1]. Decline in lung function in patients with pulmonary fibrosis is associated with mortality [2–4].

Many patients with pulmonary fibrosis experience weight loss [5–7]. Published data on the association between weight and outcomes in patients with pulmonary fibrosis are conflicting. While some studies have suggested that lower body mass index (BMI) is associated with worse outcomes [7–14], others have found no significant association [2, 5, 15–17]. Weight loss has been associated with worse outcomes in patients with pulmonary fibrosis [5, 7, 9, 10, 13, 18–21], although among overweight and obese patients, intentional weight loss may improve lung function [22, 23].

The randomized placebo-controlled INBUILD trial of nintedanib was conducted in subjects with progressive fibrosing ILDs other than idiopathic pulmonary fibrosis (IPF). The results showed that nintedanib slowed decline in lung function, with an adverse event profile characterized mainly by gastrointestinal events [24–26]. We analyzed outcomes in the INBUILD trial in subgroups by BMI at baseline and assessed associations between weight change and time-to-event outcomes using a joint modelling approach.

## Methods

The design of the INBUILD trial has been described and the protocol is publicly available [24]. Briefly, subjects had an ILD other than IPF with reticular abnormality with traction bronchiectasis (with or without honeycombing) of > 10% extent on high-resolution computed tomography (HRCT), forced vital capacity (FVC) ≥ 45% predicted and diffusing capacity of the lung for carbon monoxide (DLco) ≥ 30– < 80% predicted. Subjects met criteria for ILD progression within the prior 24 months, based on worsening of FVC, abnormalities on HRCT, or symptoms, despite management deemed appropriate in clinical practice. Subjects were randomized to receive nintedanib 150 mg bid or placebo, stratified by pattern on HRCT (usual interstitial pneumonia [UIP]-like fibrotic pattern or other fibrotic patterns [24]). Treatment interruptions (for ≤ 4 weeks for adverse events considered related to trial medication or ≤ 8 weeks for other adverse events) and dose reductions to 100 mg bid were used to manage adverse events. The trial consisted of two parts. Part A comprised 52 weeks of treatment. Part B was a variable period during which subjects continued to receive blinded treatment until all the subjects had completed the trial. The final database lock took place after all subjects had completed the follow-up visit or had entered the open-label extension study, INBUILD-ON (NCT03820726); the data available at this point are referred to as data from the whole trial.

### Analyses in subgroups by BMI at baseline

In these post-hoc analyses, we analyzed the rate of decline in FVC (mL/year) over 52 weeks in subgroups by BMI at baseline (< 25, ≥ 25 to < 30, ≥ 30 kg/m^2^). In the same subgroups, we analyzed two time-to-event endpoints: time to acute exacerbation of ILD (defined in [24]) or death and time to ILD progression (absolute decline in FVC % predicted ≥ 10%) or death. Exploratory interaction p-values were calculated to evaluate potential heterogeneity in the treatment effect of nintedanib versus placebo across the subgroups, a recommended approach for the reporting of subgroup analyses of clinical trials [27, 28]. The analyses were not adjusted for multiple testing. Adverse events are presented descriptively.

### Joint modelling

We used joint models for longitudinal and time-to-event data. These comprise two sub-models for the respective processes and an association structure to connect them. We assessed the association between change in weight (kg) and three time-to-event endpoints (time to acute exacerbation of ILD or death, time to ILD progression, time to ILD progression or death) over 52 weeks and over the whole trial. In the longitudinal sub-model, a normal mixed effects model of weight was used, with HRCT pattern (UIP-like fibrotic pattern or other fibrotic patterns) and weight at baseline as predictor variables. Separate mean slopes for subjects in the nintedanib and placebo groups were assumed. Trajectories were modelled by a linear trend with an unstructured variance–covariance matrix assumed. Weight was assessed at baseline, at weeks 2, 4, 6, 12, 24, 36 and 52 and every 16 weeks thereafter. Only values obtained before an event or censoring timepoint were considered. In the time-to-event sub‑model, a piecewise exponential model with five knots was used to model the baseline hazard. Weight was used as the endogenous time-dependent covariate and treatment as a predictor variable. The sub-model was stratified by HRCT pattern. Subjects were censored once they experienced an event and were not considered at risk of further events.

The shared parameter in each of the joint models was the estimated slope of weight (i.e., the annual rate of change in weight), which assumed that the rate of change in weight affected the risk of an event. Joint models were fitted for each time-to-event endpoint. We present the risk of the first event in terms of 1-unit, 4-unit and 10-unit decreases in weight (kg). The joint model approach was implemented with the SAS macro %JM [29]. Analyses were performed in subjects who had ≥ 1 post-baseline weight measurement and for whom data on the respective time-to-event endpoint were available.

### Characteristics of subgroups by weight loss

We present descriptive analyses of the baseline characteristics of subjects with weight loss ≤ 5% and > 5% over the whole trial, based on change in weight from baseline to any visit.

## Results

### Baseline BMI and outcomes

Among 662 subjects with available data, mean (SD) BMI at baseline was 28.3 (5.3) kg/m^2^; 28.4%, 36.6% and 35.0% of subjects had a BMI of < 25, ≥ 25 to < 30 and ≥ 30 kg/m^2^, respectively. Compared with subjects with a baseline BMI ≥ 25 to < 30 or ≥ 30 kg/m^2^, a numerically greater proportion of subjects with BMI ≤ 25 kg/m^2^ were Asian, a greater proportion had autoimmune disease-related ILDs, and a greater proportion had a UIP-like fibrotic pattern on HRCT (Additional file 1: Table S1). The mean time since diagnosis of ILD, FVC % predicted and DLco % predicted were similar across the subgroups by baseline BMI (Additional file 1: Table S1).

**Fig. 1:**
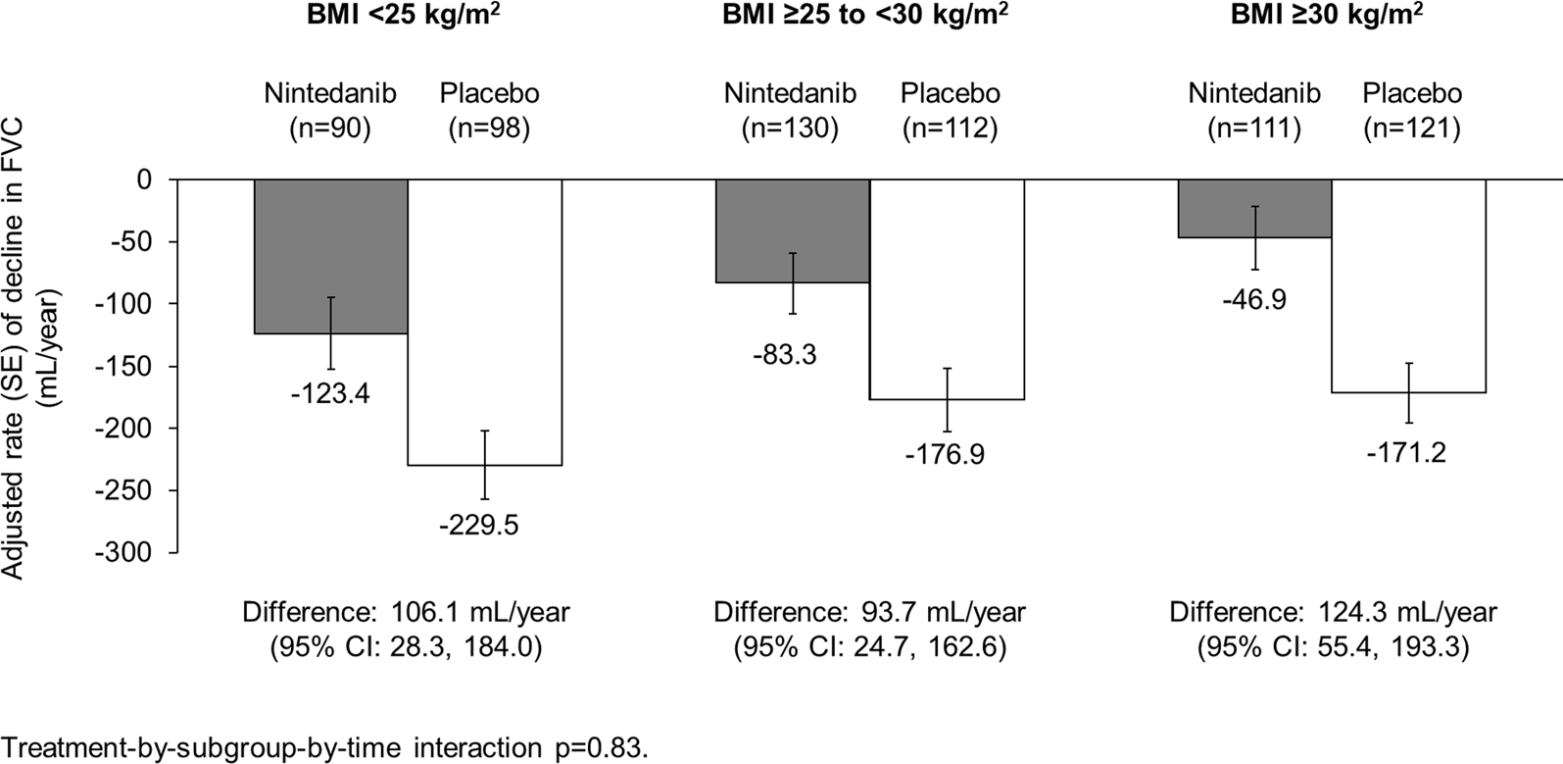
Rate of decline in FVC (mL/year) over 52 weeks by baseline BMI in the INBUILD trial

In both the nintedanib and placebo groups, the rate of decline in FVC over 52 weeks was numerically greater in subjects with baseline BMI < 25 than ≥ 25 to < 30 or ≥ 30 kg/m^2^ (Fig. 1). The exploratory interaction p-value did not indicate heterogeneity in the effect of nintedanib on reducing the rate of FVC decline among the subgroups by baseline BMI (p = 0.83). The median follow-up for the time-to-event endpoints over the whole trial was ≈19 months. In the placebo group, the proportions of subjects who had an acute exacerbation or died, or who had ILD progression or died, was greater in subjects with baseline BMI < 25 or ≥ 25 to < 30 than ≥ 30 kg/m^2^ (Table 1). The proportions of subjects with these events were similar or lower in the nintedanib than the placebo group across the subgroups by BMI, with no heterogeneity detected among the subgroups (Table 1).

**Table 1:** Outcomes over the whole INBUILD trial in subgroups by BMI at baseline

	BMI < 25 kg/m^2^		BMI ≥ 25 to < 30 kg/m^2^		BMI ≥ 30 kg/m^2^	
	Nintedanib (n = 90)	Placebo (n = 98)	Nintedanib (n = 130)	Placebo (n = 112)	Nintedanib (n = 111)	Placebo (n = 121)
Acute exacerbation or death, n (%) with event	12 (13.3)	24 (24.5)	18 (13.8)	24 (21.4)	16 (14.4)	17 (14.0)
Hazard ratio (95% CI)	0.52 (0.26, 1.04)		0.66 (0.36, 1.21)		0.92 (0.47, 1.83)	
Treatment-by-subgroup interaction	p = 0.52					
Progression of ILD^a^ or death, n (%) with event	44 (48.9)	59 (60.2)	48 (36.9)	61 (54.5)	42 (37.8)	61 (50.4)
Hazard ratio (95% CI)	0.74 (0.50, 1.10)		0.65 (0.44, 0.94)		0.64 (0.43, 0.95)	
Treatment-by-subgroup interaction	p = 0.85					

Analyzed as time to first event

*BMI* body mass index, *ILD* interstitial lung disease

^a^Absolute decline in forced vital capacity % predicted ≥ 10%

Adverse events and dose adjustments are shown in Table 2. The adverse event profile of nintedanib was similar across subgroups by BMI, with gastrointestinal adverse events the most common events. In the nintedanib group, adverse events of diarrhea and weight decrease were more frequent in subjects with baseline BMI < 25 than ≥ 25 to < 30 or ≥ 30 kg/m^2^. Decreased appetite was more frequent in subjects with baseline BMI < 25 than ≥ 25 to < 30 or ≥ 30 kg/m^2^ in both treatment groups. In the nintedanib group, adverse events leading to dose reduction were more frequent in subjects with baseline BMI < 25 than ≥ 25 to < 30 or ≥ 30 kg/m^2^. Adverse events led to treatment discontinuation more frequently in subjects with baseline BMI < 25 than ≥ 25 to < 30 or ≥ 30 kg/m^2^ in both treatment groups. The most frequent adverse event leading to discontinuation of nintedanib was diarrhea, which occurred at rates of 4.3, 6.1 and 3.9 events per 100 patient-years in subjects with baseline BMI < 25, ≥ 25 to < 30 and ≥ 30 kg/m^2^, respectively. One subject in the nintedanib group (with baseline BMI < 25 kg/m^2^) and one subject in the placebo group (with baseline BMI ≥ 25 to < 30 kg/m^2^) discontinued treatment due to weight loss.

**Table 2:** Adverse events over the whole INBUILD trial in subgroups by BMI at baseline

	BMI < 25 kg/m^2^				BMI ≥ 25 to < 30 kg/m^2^				BMI ≥ 30 kg/m^2^			
	Nintedanib (n = 90)		Placebo (n = 98)		Nintedanib (n = 130)		Placebo (n = 112)		Nintedanib (n = 111)		Placebo (n = 121)	
	n (%)	Rate per 100 patient-years	n (%)	Rate per 100 patient-years	n (%)	Rate per 100 patient-years	n (%)	Rate per 100 patient-years	n (%)	Rate per 100 patient-years	n (%)	Rate per 100 patient-years
Any adverse event(s)	89 (98.9)	1048.3	95 (96.9)	435.8	127 (97.7)	589.3	104 (92.9)	288.4	109 (98.2)	716.1	109 (90.1)	281.7
Most frequent adverse events^a^												
Diarrhea	71 (78.9)	199.2	25 (25.5)	23.3	92 (70.8)	130.9	29 (25.9)	23.3	77 (69.4)	112.8	31 (25.6)	22.5
Nausea	27 (30.0)	30.7	9 (9.2)	6.9	39 (30.0)	30.5	11 (9.8)	7.4	33 (29.7)	30.5	13 (10.7)	8.2
Vomiting	19 (21.1)	18.2	3 (3.1)	2.2	21 (16.2)	14.9	5 (4.5)	3.3	24 (21.6)	19.4	8 (6.6)	4.8
Decreased appetite	21 (23.3)	22.1	9 (9.2)	6.8	18 (13.8)	11.9	7 (6.3)	4.6	15 (13.5)	10.8	7 (5.8)	4.2
Nasopharyngitis	19 (21.1)	19.9	23 (23.5)	20.2	21 (16.2)	14.3	14 (12.5)	9.5	14 (12.6)	9.7	11 (9.1)	6.9
Dyspnea	14 (15.6)	13.0	11 (11.2)	8.4	19 (14.6)	12.6	27 (24.1)	19.4	19 (17.1)	13.4	19 (15.7)	12.0
Bronchitis	12 (13.3)	11.2	19 (19.4)	15.5	21 (16.2)	14.4	20 (17.9)	14.3	15 (13.5)	10.7	25 (20.7)	16.4
Weight decrease	19 (21.1)	18.8	7 (7.1)	5.2	18 (13.8)	12.0	8 (7.1)	5.2	12 (10.8)	8.5	3 (2.5)	1.7
ALT increased	14 (15.6)	13.9	2 (2.0)	1.5	18 (13.8)	11.9	5 (4.5)	3.2	17 (15.3)	12.0	6 (5.0)	3.6
Cough	8 (8.9)	7.2	9 (9.2)	6.9	15 (11.5)	9.8	12 (10.7)	8.1	17 (15.3)	12.1	30 (24.8)	20.8
Progression of ILD^b^	9 (10.0)	8.1	27 (27.6)	22.3	12 (9.2)	7.3	17 (15.2)	11.1	7 (6.3)	4.6	12 (9.9)	7.2
AST increased	15 (16.7)	15.2	2 (2.0)	1.5	13 (10.0)	8.5	4 (3.6)	2.6	15 (13.5)	10.3	7 (5.8)	4.2
Adverse event(s) leading to treatment discontinuation	27 (30.0)	24.0	18 (18.4)	13.2	27 (20.8)	16.6	15 (13.4)	9.5	19 (17.1)	12.4	15 (12.4)	8.8
Adverse event(s) leading to dose reduction	44 (48.9)	63.8	4 (4.1)	3.0	46 (35.4)	40.0	2 (1.8)	1.3	34 (30.6)	28.2	9 (7.4)	5.4
Serious adverse event(s)^c^	42 (46.7)	46.4	57 (58.2)	56.0	59 (45.4)	44.2	57 (50.9)	45.4	46 (41.4)	36.7	50 (41.3)	35.0
Fatal adverse event(s)	4 (4.4)	3.4	12 (12.2)	8.7	10 (7.7)	6.0	16 (14.3)	10.1	7 (6.3)	4.5	8 (6.6)	4.6

Based on adverse events reported between the first trial drug intake and 28 days after the last trial drug intake

*ALT* alanine aminotransferase, *AST* aspartate aminotransferase, *BMI* body mass index, *MedDRA* Medical Dictionary for Regulatory Activities

^a^Adverse events were coded based on preferred terms in the MedDRA version 22.0. Adverse events with an incidence rate of >10 events per 100 patient-years in either treatment group in the overall population are shown

^b^Corresponded to the MedDRA preferred term “interstitial lung disease”

^c^Event that resulted in death, was life-threatening, resulted in hospitalization or prolonged hospitalization, resulted in persistent or clinically significant disability or incapacity, was a congenital anomaly or birth defect, or was deemed serious for any other reason

### Weight loss and outcomes

Subjects in the nintedanib group had a significantly greater decrease in weight than subjects in the placebo group over 52 weeks (Additional file 1: Table S2) and over the whole trial (Table 3). The baseline characteristics of the subgroups of subjects by weight loss ≤ 5% and > 5% over the whole trial were similar (Additional file 1: Table S3).

**Table 3:** Association between change in weight (slope) and risk of outcomes over the whole INBUILD trial

	Acute exacerbation or death	ILD progression	ILD progression or death
*N included*			
Placebo	330	327	327
Nintedanib	331	326	326
Longitudinal sub-model^a^			
Estimated change in weight (kg) (95% CI) with placebo	− 1.60 (− 2.08, − 1.12)	− 0.76 (− 1.34, − 0.19)	− 0.93 (− 1.50, − 0.37)
Estimated change in weight (95% CI) difference for nintedanib vs placebo	− 1.51 (− 2.19, − 0.84)	− 2.07 (− 2.84, − 1.31)	− 2.00 (− 2.76, − 1.24)
p-value	< 0.001	< 0.001	< 0.001
Time-to-event sub-model^b^			
n (%) with event			
Placebo	64 (19.4)	156 (47.7)	177 (54.1)
Nintedanib	46 (13.9)	109 (33.4)	129 (39.6)
Hazard ratio (95% CI) for nintedanib vs placebo	0.60 (0.41, 0.89)	0.68 (0.52, 0.89)	0.64 (0.50, 0.81)
p-value	0.011	0.004	< 0.001
Association between change in weight (slope) and risk of event, hazard ratio (95% CI)			
Per 1 kg decrease	1.08 (1.03, 1.14)	0.96 (0.91, 1.01)	1.00 (0.96, 1.04)
Per 4 kg decrease	1.38 (1.13, 1.68)	0.83 (0.68, 1.02)	1.01 (0.87, 1.18)
Per 10 kg decrease	2.23 (1.36, 3.65)	0.64 (0.39, 1.05)	1.03 (0.70, 1.53)
p-value	0.002	0.08	0.88

*UIP* usual interstitial pneumonia, *ILD* interstitial lung disease

^a^Random effects normal linear model of weight (kg) with HRCT pattern (UIP-like pattern or other fibrotic patterns) and weight at baseline as predictor variables, a separate slope assumed for the nintedanib and placebo groups, and trajectories modelled by a linear trend with an unstructured variance–covariance matrix assumed

^b^Proportional hazard model with a piecewise exponential baseline hazard, stratified by HRCT pattern, with treatment as a predictor variable and the endogenous time-dependent covariate of weight (kg) as estimated slope of the longitudinal response

**Fig. 2:**
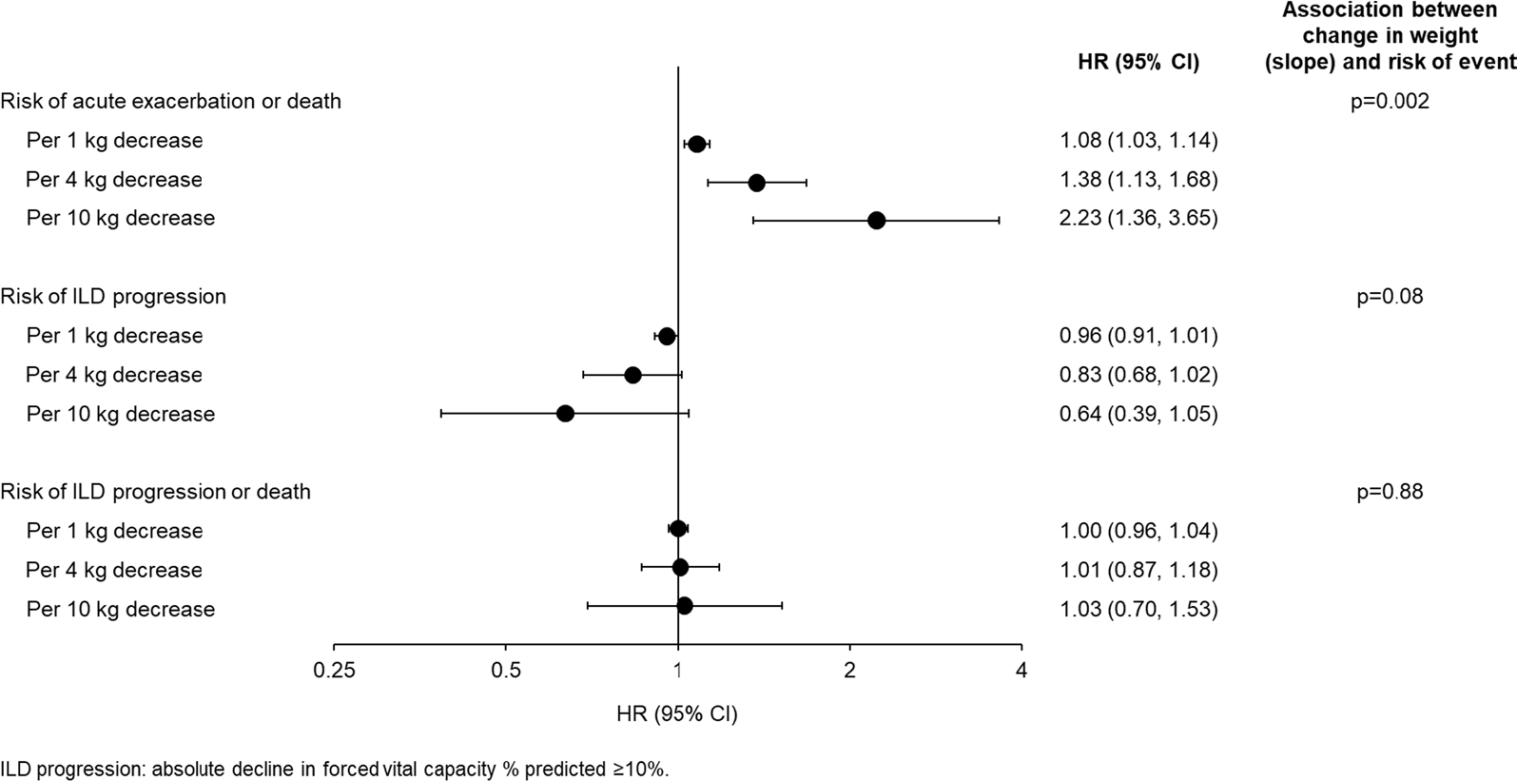
Association between change in weight (slope) and risk of outcomes over the whole INBUILD trial

Over the whole trial, 19.4% of subjects in the placebo group and 13.9% of subjects in the nintedanib group had an acute exacerbation or died. There was a significant association between weight decrease and time to acute exacerbation or death over 52 weeks (Additional file 1: Table S2) and over the whole trial (Table 3). Based on the estimated slope, a 4 kg weight decrease corresponded to a 1.38-fold (95% CI 1.13, 1.68) increase in the risk of acute exacerbation or death (Fig. 2).

Over the whole trial, 47.7% of subjects in the placebo group and 33.4% of subjects in the nintedanib group experienced ILD progression, and 54.1% of subjects in the placebo group and 39.6% of subjects in the nintedanib group experienced ILD progression or death. No association was detected between weight decrease and the risk of ILD progression or the risk of ILD progression or death (Table 3, Fig. 2 and Additional file 1: Table S2).

## Discussion

These analyses of data from the INBUILD trial suggest that there may be associations between baseline BMI or weight loss and clinically relevant outcomes in patients with PPF. The rate of FVC decline, and the risk of ILD progression or death, were numerically greater in subjects with baseline BMI < 25 kg/m^2^ than in those with higher BMI. Weight loss during the trial was associated with a significantly increased risk of acute exacerbation or death. As observed in clinical trials in patients with other ILDs [10, 30], nintedanib had a consistent effect on slowing the progression of ILD across the subgroups by baseline BMI.

Our finding that the rate of decline in FVC was greatest in subjects with baseline BMI < 25 kg/m^2^ is consistent with observations in subjects with IPF in the INPULSIS trials [10] and other studies in subjects with IPF and systemic sclerosis-associated ILD [13, 14]. In an analysis including data from trials of pirfenidone in patients with IPF, the annualized decline in FVC % predicted was greater in patients with baseline BMI < 25 kg/m^2^ than BMI ≥ 25 to < 30 or ≥ 30 kg/m^2^ in the placebo groups, but this was not observed in patients who received pirfenidone [13]. Our finding that the risk of ILD progression or death was numerically greater in subjects with baseline BMI < 25 kg/m^2^ is consistent with observations from previous studies showing higher mortality in patients with pulmonary fibrosis who have lower BMI [7–9, 12]. The reasons why low BMI is associated with worse outcomes in patients with ILDs are not understood, but may be related to loss of muscle mass [31, 32] or to increased levels of pro-inflammatory cytokines, such as tumor necrosis factor, which have been associated with weight loss in animal studies [33].

Various approaches can be used to investigate associations between a longitudinal measure such as weight and the risk of an outcome. In these analyses, we used a joint modelling approach, as this enables longitudinal markers and time-to-event endpoints to be analyzed simultaneously, overcoming issues of bias and measurement error that occur when repeated measurements and outcomes are analyzed separately, when analyses are based on post-baseline subgroups, or when analyses do not consider longitudinal endpoints as endogenous time-varying factors [34–37]. This joint modelling also enabled us to evaluate the validity of weight change as a surrogate endpoint for the time-to-event endpoints according to the three levels of surrogacy defined by Taylor and Elston [38]. The significant association between the outcome of weight change and the risk of acute exacerbation or death fulfils Taylor and Elston’s criteria for surrogacy at level two, but further validation is required. While several previous studies have shown that weight loss is associated with a greater risk of mortality in patients with pulmonary fibrosis [5, 7, 9, 10, 13, 18–21], we are not aware of prior studies suggesting an association between weight loss and acute exacerbations of ILD.

The adverse event profile of nintedanib was generally similar across the subgroups by baseline BMI, but adverse events of diarrhea, decreased appetite and weight decrease, and adverse events leading to dose reduction and treatment discontinuation, were more frequent in subjects who had a baseline BMI < 25 kg/m^2^ than a higher BMI. In the INPULSIS, SENSCIS and INBUILD trials in subjects with pulmonary fibrosis, the proportion of subjects with adverse events of weight loss over 52 weeks ranged from 9.7 to 12.3% in the nintedanib groups compared to 3.3 to 4.2% in the placebo groups [6, 24, 39]. The reported proportion of patients with IPF treated with nintedanib who experience weight loss in real-world studies is highly variable, likely reflecting differences in methodology and the patient populations studied [40–42]. Clinicians should be aware of weight loss as a potential adverse event of nintedanib, particularly in patients with low BMI, and consider nutritional interventions when required. Management of gastrointestinal adverse events associated with nintedanib therapy using symptomatic therapies such as loperamide and dose adjustment is important to minimize their impact and help patients remain on treatment [43, 44].

Strengths of our analyses include the robust collection of data on FVC and weight in the setting of a clinical trial, and the use of a joint modelling approach to assess the associations between weight loss and outcomes [34–37]. Limitations include that BMI is limited as a measure of nutritional status [45, 46] and that the subgroups with different BMI differed in factors beyond BMI. The lowest BMI subgroup included a greater proportion of subjects with autoimmune disease-related ILDs, which are associated with gastrointestinal complications that may lead to weight loss, and this may have influenced the risk of outcomes across the subgroups. The numbers of subjects with individual ILD diagnoses were too small for these subgroups to be analyzed separately. The number of subjects who were underweight was too small for this group to be analyzed separately. The number of acute exacerbations available for use in the time-to-event analyses was quite small. There were too few deaths for associations between weight change and death alone to be analyzed. Our analyses did not establish cause and effect. These analyses were post-hoc and should be considered exploratory.

## Conclusions

In conclusion, these analyses of data from the INBUILD trial suggest that in subjects with PPF, lower BMI at baseline and weight loss may be associated with worse outcomes. Nintedanib had a consistent effect on slowing ILD progression across subgroups by baseline BMI. Physicians should monitor weight in patients with PPF and consider interventions where necessary.

## Supplementary Information

**Additional file 1: Table S1.** Baseline characteristics in subgroups by body mass index (BMI) in the INBUILD trial. **Table S2.** Association between change in weight (slope) and risk of outcomes over 52 weeks in the INBUILD trial. **Table S3.** Baseline characteristics of subgroups of subjects by weight loss ≤5% and >5% over the whole INBUILD trial.

## Data Availability

To ensure independent interpretation of clinical study results and enable authors to fulfil their role and obligations under the ICMJE criteria, BI grants all external authors access to relevant clinical study data. In adherence with the BI Policy on Transparency and Publication of Clinical Study Data, scientific and medical researchers can request access to clinical study data after publication of the primary manuscript in a peer-reviewed journal, regulatory activities are complete and other criteria are met. Researchers should use https://vivli.org/ to request access to study data and visit https://www.mystudywindow.com/msw/datasharing for further information.
