# Supplementary material for: Weight loss and outcomes in subjects with progressive pulmonary fibrosis: data from the INBUILD trial
Source: Respir Res. 2023 Mar 9;24:71. doi: 10.1186/s12931-023-02371-z (PMC9999543; doi:10.1186/s12931-023-02371-z)
Supplement: Supplementary file 1 — Additional file 1: Table S1. Baseline characteristics in subgroups by body mass index (BMI) in the INBUILD trial. Table S2. Association between change in weight (slope) and risk of outcomes over 52 weeks in the INBUILD trial. Table S3. Baseline characteristics of subgroups of subjects by weight loss ≤5% and >5% over the whole INBUILD trial. [file 12931_2023_2371_MOESM1_ESM.docx]

**Weight loss and outcomes in subjects with progressive pulmonary fibrosis: data from the INBUILD trial**

**Additional file 1**

**Table S1**. Baseline characteristics in subgroups by body mass index (BMI) in the INBUILD trial.

|  | **BMI <25 kg/m^2^**  **(n=188)** | **BMI ≥25 to <30 kg/m^2^**  **(n=242)** | **BMI ≥30 kg/m^2^**  **(n=232)** |
| --- | --- | --- | --- |
| Male, n (%) | 93 (49.5) | 148 (61.2) | 114 (49.1) |
| Age (years), mean (SD) | 65.7 (10.7) | 66.6 (9.8) | 64.9 (8.9) |
| Weight (kg), mean (SD) | 59.8 (9.2) | 75.5 (10.5) | 92.4 (14.4) |
| BMI (kg/m^2^), mean (SD) | 22.5 (1.9) | 27.4 (1.5) | 33.9 (3.9) |
| Current or former smoker, n (%) | 83 (44.1) | 131 (54.1) | 124 (53.4) |
| Race, n (%)* |  |  |  |
| White | 94 (50.0) | 177 (73.1) | 216 (93.1) |
| Asian | 93 (49.5) | 60 (24.8) | 10 (4.3) |
| Black/African-American | 1 (0.5) | 5 (2.1) | 4 (1.7) |
| American Indian/Alaska Native/Native Hawaiian/other Pacific Islander | 0 | 0 | 1 (0.4) |
| Years since diagnosis of ILD, mean (SD)^†^ | 4.3 (4.5) | 3.6 (3.3) | 3.4 (3.4) |
| UIP-like fibrotic pattern on HRCT, n (%) | 133 (70.7) | 149 (61.6) | 129 (55.6) |
| FVC % predicted, mean (SD) | 69.8 (16.9) | 69.2 (15.2) | 68.1 (15.0) |
| DLco % predicted^‡^, mean (SD) | 44.0 (12.8) | 45.9 (12.3) | 48.1 (15.3) |
| ILD diagnosis, n (%) |  |  |  |
| Hypersensitivity pneumonitis | 35 (18.6) | 57 (23.6) | 81 (34.9) |
| Autoimmune ILDs | 61 (32.4) | 60 (24.8) | 49 (21.1) |
| Idiopathic non-specific interstitial pneumonia | 32 (17.0) | 47 (19.4) | 46 (19.8) |
| Unclassifiable IIP | 38 (20.2) | 43 (17.8) | 32 (13.8) |
| Other ILDs^§^ | 22 (11.7) | 35 (14.5) | 24 (10.3) |

*Data from subjects who selected one race. One subject ticked more than one box. ^†^Information was not available for one subject. ^‡^Corrected for haemoglobin. Information was not available for 8 subjects. ^§^Included sarcoidosis, exposure-related ILDs and other terms in the “Other fibrosing ILD” category of the case report form. DLco; diffusing capacity of the lung for carbon monoxide; FVC, forced vital capacity; HRCT, high-resolution computed tomography; IIP, idiopathic interstitial pneumonia; ILD, interstitial lung disease; UIP, usual interstitial pneumonia.

**Table S2.** Association between change in weight (slope) and risk of outcomes over 52 weeks in the INBUILD trial.

|  | **Acute exacerbation or death** | **ILD progression** | **ILD progression or death** |
| --- | --- | --- | --- |
| N included |  |  |  |
| Placebo | 330 | 327 | 327 |
| Nintedanib | 331 | 326 | 326 |
| **Longitudinal sub-model*** |  |  |  |
| Estimated change in weight (kg) (95% CI) with placebo | −1.44 (−1.99, −0.89) | −0.75 (−1.39, −0.11) | −0.79 (−1.44, −0.15) |
| Estimated change in weight (95% CI) difference for nintedanib vs placebo | −1.98 (−2.75, −1.21) | −2.44 (−3.30, −1.58) | −2.42 (−3.28, −1.56) |
| p-value | <0.001 | <0.001 | <0.001 |
| **Time-to-event sub-model**^†^ |  |  |  |
| n (%) with event |  |  |  |
| Placebo | 32 (9.7) | 111 (33.9) | 120 (36.7) |
| Nintedanib | 26 (7.9) | 67 (20.6) | 79 (24.2) |
| Hazard ratio (95% CI) for nintedanib vs placebo | 0.68 (0.39, 1.17) | 0.68 (0.49, 0.94) | 0.66 (0.49, 0.89) |
| p-value | 0.16 | 0.019 | 0.007 |
| **Association between change in weight (slope) and risk of event, hazard ratio (95% CI)** |  |  |  |
| Per 1 kg decrease | 1.08 (1.02, 1.15) | 0.97 (0.92, 1.01) | 0.98 (0.93, 1.03) |
| Per 4 kg decrease | 1.37 ( 1.08, 1.73) | 0.87 ( 0.72, 1.06) | 0.92 ( 0.76, 1.11) |
| Per 10 kg decrease | 2.18 (1.20, 3.96) | 0.71 (0.43, 1.14) | 0.81 (0.50, 1.30) |
| p-value | 0.011 | 0.16 | 0.38 |

*Random effects normal linear model of weight (kg) with HRCT pattern (UIP-like pattern or other fibrotic patterns) and weight at baseline as predictor variables, a separate slope assumed for the nintedanib and placebo groups, and trajectories modelled by a linear trend with an unstructured variance–covariance matrix assumed. ^†^Proportional hazard model with a piecewise exponential baseline hazard, stratified by HRCT pattern, with treatment as a predictor variable and the endogenous time-dependent covariate of weight (kg) as estimated slope of the longitudinal response. UIP, usual interstitial pneumonia. ILD, interstitial lung disease.

**Table S3.** Baseline characteristics of subgroups of subjects by weight loss ≤5% and >5% over the whole INBUILD trial.

|  | **Weight loss ≤5%** | | **Weight loss >5%** | |
| --- | --- | --- | --- | --- |
|  | **Nintedanib (n=149)** | **Placebo (n=202)** | **Nintedanib (n=181)** | **Placebo (n=129)** |
| Male, n (%) | 86 (57.7) | 108 (53.5) | 93 (51.4) | 69 (53.5) |
| Age (years), mean (SD) | 64.4 (10.0) | 65.9 (9.9) | 66.0 (9.5) | 66.9 (9.7) |
| Weight (kg), mean (SD) | 78.2 (17.8) | 75.9 (16.9) | 76.1 (16.1) | 78.7 (19.5) |
| BMI (kg/m^2^), mean (SD) | 28.2 (5.2) | 28.0 (4.8) | 28.1 (5.0) | 29.0 (6.4) |
| Current or former smoker, n (%) | 78 (52.3) | 101 (50.0) | 91 (50.3) | 68 (52.7) |
| Race*, n (%) |  |  |  |  |
| White | 112 (75.2) | 153 (75.7) | 128 (70.7) | 93 (72.1) |
| Asian | 34 (22.8) | 47 (23.3) | 49 (27.1) | 33 (25.6) |
| Black/African-American | 2 (1.3) | 2 (1.0) | 3 (1.7) | 3 (2.3) |
| American Indian/Alaska Native/Native Hawaiian/other Pacific Islander | 0 | 0 | 1 (0.6) | 0 |
| Years since diagnosis of ILD, mean (SD)^†^ | 3.5 (3.5) | 4.1 (3.8) | 3.6 (3.9) | 3.6 (3.5) |
| UIP-like fibrotic pattern on HRCT, n (%) | 91 (61.1) | 127 (62.9) | 115 (63.5) | 79 (61.2) |
| FVC % predicted, mean (SD) | 69.1 (15.6) | 69.9 (14.7) | 68.5 (16.5) | 68.4 (16.0) |
| DLco % predicted^‡^, mean (SD) | 44.2 (11.2) | 48.9 (15.1) | 44.5 (12.5) | 46.3 (14.7) |
| ILD diagnosis, n (%) |  |  |  |  |
| Hypersensitivity pneumonitis | 36 (24.2) | 58 (28.7) | 48 (26.5) | 31 (24.0) |
| Autoimmune disease-related ILDs | 37 (24.8) | 54 (26.7) | 43 (23.8) | 34 (26.4) |
| Idiopathic non-specific interstitial pneumonia | 34 (22.8) | 35 (17.3) | 30 (16.6) | 26 (20.2) |
| Unclassifiable IIP | 23 (15.4) | 28 (13.9) | 41 (22.7) | 22 (17.1) |
| Other ILDs^§^ | 19 (12.8) | 27 (13.4) | 19 (10.5) | 16 (12.4) |

*Data from subjects who selected one race. One subject ticked more than one box. ^†^Information was not available for one subject. ^‡^Corrected for hemoglobin. Information was not available for 8 subjects. ^§^Included sarcoidosis, exposure-related ILDs and other terms in the “Other fibrosing ILD” category of the case report form. DLco; diffusing capacity of the lung for carbon monoxide; FVC, forced vital capacity; HRCT, high-resolution computed tomography; IIP, idiopathic interstitial pneumonia; ILD, interstitial lung disease; UIP, usual interstitial pneumonia.
